# Community-based mental health centers in Ukraine — protocol for a mixed methods evaluation study using the RE-AIM framework

**DOI:** 10.3389/fpubh.2026.1740963

**Published:** 2026-03-27

**Authors:** Maha Eid Madkour, Ann-Kathrin Schwientek, Viktoriia Yasenok, Anja Frei, Marco Kaufmann, Viktoriia Petrashenko, Miquel Serra Burriel, Svitlana Piven, Natalia Kolyada, Erich Seifritz, Mariya Bachmaha, Orest Suvalo, Viktor Von Wyl, Milo A. Puhan, Andriana Kostenko

**Affiliations:** 1 Epidemiology, Biostatistics and Prevention Institute, University of Zurich, Zurich, Switzerland; 2 Population Research Center, University of Zurich, Zurich, Switzerland; 3 Sumy State University, Sumy, Ukraine; 4 Department of Adult Psychiatry and Psychotherapy, University Hospital of Psychiatry Zurich, Zurich, Switzerland; 5 Mental Health for Ukraine Project (MH4U), Lviv, Ukraine; 6 Institute of Mental Health, Ukrainian Catholic University, Lviv, Ukraine; 7 Institute for Implementation Science in Health Care, University of Zurich, Zurich, Switzerland

**Keywords:** community-based care, evaluation, mental health centers, mental health service, mixed-methods, RE-AIM, real-world data, Ukraine

## Abstract

**Background:**

Russia’s full-scale invasion of Ukraine has deepened the strain on an already fragile mental health system, long shaped by Soviet institutionalized psychiatry. In response, Ukraine has started a national reform to modernize mental health services by shifting from an institutional to a community-based mental health care model. Implementation of his transition is supported by the international technical project Mental Health for Ukraine (MH4U).

**Aims:**

To generate evidence-based evaluation of the community-based mental health centers as a basis for continued improvement and to guide national scale-up.

**Methods and analysis:**

We will conduct a mixed-methods evaluation guided by the RE-AIM framework. First, quantitative data will be collected through (a) questionnaires administered to patient’s prospective longitudinal cohort at baseline, three, and six months, (b) questionnaires from providers at the baseline and in the follow-up, and (c) administrative records. Second, qualitative data will be gathered through (a) interviews with subsample of participating patients and all the providers, and (b) focus groups with community stakeholders for each oblast (administrative region). Analyses will follow the RE-AIM framework. Adhering to a learning health systems (LHS) framework, we established continuous feedback loops and workshops to communicate study findings to MH4U implementers and stakeholders in Ukraine and Switzerland.

**Conclusion:**

Evaluation will provide unique evidence on not only whether the community-based Mental Health Center model works, but also how and why it works in real-world conditions. Findings will also inform national policy on mental health reform and offer lessons for global mental health systems operating under crisis conditions.

**Clinical trial registration:**

https://www.isrctn.com/ISRCTN61471084, ISRCTN61471084.

## Background

The ongoing war in Ukraine is expected to affect the mental health of Ukrainian citizens for decades (1). Ukraine estimated population is 39 million in 2025 (2). In 2023, the World Health Organisation estimates that approximately 9.6 million people in Ukraine are at risk of, or living with, a mental health condition, and 3.9 million with moderate to severe symptoms (3). Furthermore, the Ukrainian Ministry of Health predicts that 4 million people will need psychotropic medication, and up to 15 million will need psychosocial support (4).

Ukraine inherited an institutionalized mental health care system shaped by the Soviet legacy (5). This history has contributed to enduring mistrust and strong stigma surrounding mental health care (6, 7). Nevertheless, the Ukrainian health system long struggled with the lack of clear support pathways for individuals with mental health problems, resulting in delayed access to adequate diagnosis and treatment (5, 8). Social services for people with mental disorders in the community are limited or even absent (9).

Recognizing this historic context, in 2017, the Cabinet of Ministers of Ukraine approved a key strategic document for the reform of the mental health care system: “The Concept Note on Mental Health Care Development in Ukraine”. The Concept Note mandated state-sponsored and international initiatives to modernize and decentralize mental health care in Ukraine (10). A central element of this reform is the transition away from institutionalized social care facilities (internats) toward the community-based Mental Health Center (MHC) model to allow people to receive care while remaining active in their communities (11). MHCs have been shown globally to be an evidence-based model to improve access to care, health, and social inclusion while reducing stigma (12–14). Thus, the Ukrainian government commits to establishing MHCs across the country. MHC provides specialized mental health care alongside non-medicalized psychosocial support for victims of the war and the wider community (15).

International collaborative initiatives have been established to advance the transition to MHCs in Ukraine (16). One key initiative, the Mental Health for Ukraine Project (MH4U) was launched before the 2022 invasion in collaboration with the Ukrainian Catholic University’s Institute of Mental Health and supported by the Swiss Agency for Development and Cooperation (DEZA) (16, 17). MH4U embodies the approaches outlined in the “Concept for the Development of Mental Health Care in Ukraine until 2030.” The concept aims to create a comprehensive and effective mental health care system (8, 18). MH4U provides MHCs with financial support, infrastructure development, training, and implementation guidance in alignment with Ukraine’s national mental health reform (15, 16).

MHCs serve three complementary roles. First, they provide direct mental health services in outpatient and day-clinic settings. Services are delivered by interdisciplinary teams of providers, i.e., psychiatrists, psychologists, psychotherapists, nurses, and social workers. Second, they act as a community hub for care coordination, managing referrals, and collaboration between medical and social service providers who work in alignment with the community to reduce stigma and improve mental health literacy. Third, they engage communities through public awareness, low-threshold prevention, and education initiatives (16).

Despite the potential of MHCs, no studies have evaluated how these MHCs operate in real-world conditions, their capacity to develop and expand, or their integration into communities, especially during wartime. Achieving the full potential of this model requires continuous learning, coordination, and improvement across all levels of the mental health care system.

### Aims

We aim to establish an evidence-based evaluation of the MHC model to guide its refinement and national scale-up in Ukraine.

- Measure the real-world implementation and identify potential improvement variables across three levels: community, provider, and patient.
- Examine how collaboration across the multi-level system affects outcomes.
- Deliver iterative feedback to stakeholders to support continuous quality improvement and inform policy decisions for the expansion of MHCs.

## Methods and analysis

### Design

**Figure 1:**
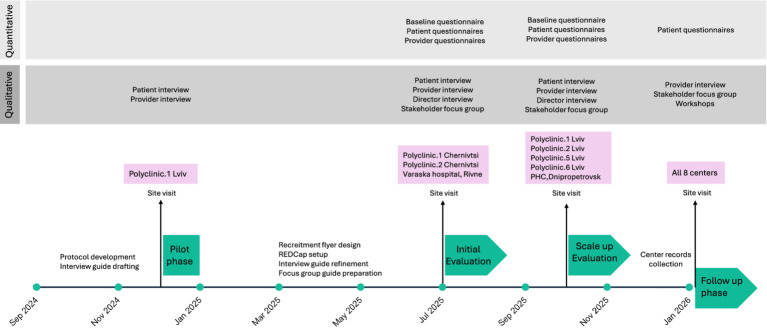
Overview of study phases, sites, and timeline. Timeline representing study phases, including participating MHCs across four oblasts, measurement and data collection.

**Figure 2:**
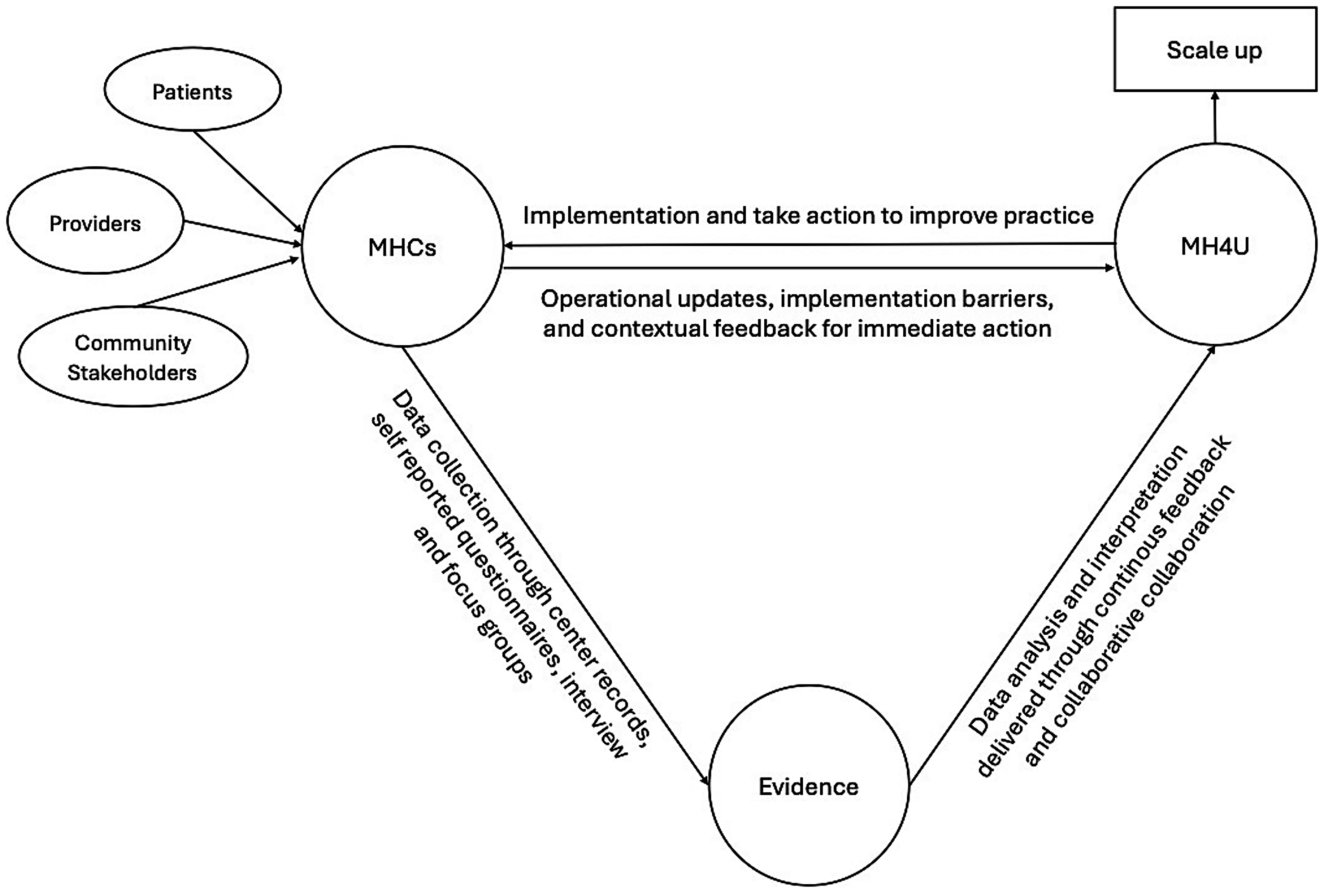
Learning health system framework for the MHCs evaluation. Figure represent the LHS applied to MHCs evaluation to produce evidence. The figure illustrate how interpreted data contributes to iterative cycles of knowledge generation, evidence synthesis, and improvement in MHC.

We employ a mixed-methods evaluation design guided by the RE-AIM (Reach, Effectiveness, Adoption, Implementation, and Maintenance) framework (19, 20). This framework allows assessment at both individual and organizational levels while capturing both internal and external validity, making it well-suited for evaluating real-world interventions and guiding scale-up efforts (19). Figure 1 shows the phases, sites, and timelines of the study. Consistent with the Learning Health System (LHS) approach, data generated through the evaluation will be continuously fed back to the MH4U team and stakeholders to inform real-time adaptation and improvement (Figure 2) (21).

### Study setting and participants

#### MHCs

We will conduct the study across the current eight MHCs and their respective communities, including four in Lviv, two in Chernivtsi, one in Dnipropetrovsk, and one in Rivne. These oblasts may differ in the mental health impact of the war, providing important contextual variation for the evaluation. Mostly, MHCs are structural components of policlinics, i.e., outpatient services. However, in Dnipro, MHC is a part of the Primary Health Care (PHC) service. While in Rivne, MHC is a hospital department within a cluster hospital, providing a network of medical services to more than one community.

### Participants

#### Patients

##### Intervention cohort

The inclusion criteria are: (a) Adults (≥18 years), (b) have received one to three treatment sessions at one of the participating MHCs, and (c) have provided informed consent. Patients will be excluded if they have only received an emergency session or if a treating psychiatrist determines they have severe cognitive dysfunction or an acute mental health condition that would impair their ability to participate.

##### Sample size calculation

We will recruit a prospective longitudinal cohort via a non-probability sampling technique. Each participating MHC representing a cluster because patient outcomes are expected to be correlated within centers due to shared staff, resources and local community. Approximately 400 patients for the 8 MHCs (targeting ~50 per MHC) will be enrolled in the quantitative assessment. The primary outcome is the change in depressive symptoms measured by the Patient Health Questionnaire-9 (PHQ-9) scale from baseline to follow up 4 (approximately one year) (22).

We estimated the variance of the PHQ-9 change score empirically as:


$$\mathrm{Var}\;(\Delta )=\sigma_{b}^{2}+\sigma_{f}^{2}-2r\sigma_{b}\sigma_{f}\;$$

Where $\sigma_{b}=5.97$ and $\sigma_{f}=5.50$ are the baseline and follow-up 4 standard deviations (SDs), respectively, and r=0.546 is the within-person correlation. Substituting these values gives Var(Δ)≈30.0 , yielding ${\mathrm{SD}}_{\Delta }\approx 5.48$ .

We account for clustering between MHCs using design effect,


DE=1+(m−1)ICC

Where m = 50 is the average cluster size and ICC is the intraclass correlation. Under this framework (two-sided significance level 5 and 80% power), the minimum detectable difference in difference (DiD) effect in PHQ-9 points is approximately 0.94, 1.08, and 1.43 for ICC = 0.01, 0.02, and 0.05, respectively.

We will invite a subsample from the quantitative cohort of patients to interviews through a purposive sampling strategy (23). Patients will be selected with varying treatment exposure (≥1 session) to capture diverse treatment experiences and outcomes. The qualitative sample size will be guided by content saturation.

##### The external control cohort

The external control data will be drawn from the Mental Health Assessment of the Ukrainian Population (MAP) studies, a large-scale cohort designed to better understand the mental stress experienced by the Ukrainian population as a result of the war (24, 25). MAP participants include adults aged ≥18 years across multiple oblasts in Ukraine. They were recruited using probability-based sampling to ensure representativeness of the general population.

#### Providers

All providers will be recruited, given the limited and well-defined staff composition at each MHC, for both the quantitative and qualitative components.

Given the ongoing war, we will recruit a control group of providers to enhance quantitative data interpretability. This control group will include all providers from eight MHCs that are not supported by MH4U.

#### Community stakeholders

We will recruit six to nine community stakeholders for focus groups from two groups: (a) medical specialists (GPs, neurologists, rehabilitation specialists), (b) non-medical (local health authorities, NGOs, and social workers), who cooperate or partner with the selected MHCs.

### RE-AIM operationalization

Tables 1–5 shows the RE-AIM key indicators and data sources we will use to address the RE-AIM dimensions. We will assess the five dimensions of the RE-AIM framework across three levels (patient, provider, and community) as follows:

1. 1 Reach

- Quantitative assessment- patient level
  We will capture the number, proportion, demographics, clinical characteristics, and referral sources of patients receiving care at MHCs.
- Qualitative assessment- patient level
  It will complement the quantitative data to identify barriers and facilitators to accessing services.

2 Effectiveness

- Quantitative assessment - patient level
  We will assess patient symptoms and severity of specific mental health conditions using standardized questionnaires previously applied in the MAP studies, covering Post-Traumatic Stress Disorder (PTSD), depression, somatic distress syndrome, anxiety, and alcohol use, in addition to health-related quality of life and mental well-being. (24, 25)
- Qualitative assessment - patient level
  To understand patient perspectives on perceived benefits, harms, and contextual explanations for outcomes.

3 Adoption

- Quantitative assessment- provider level
  We will capture the characteristics of all eight MHCs and their affiliated providers to quantify the extent and representativeness of adoption. We will assess factors influencing adoption. We will also measure work-and non-work-related health factors to control for external confounders in this war-affected context.
- Qualitative assessment- providers and community levels
  We will examine providers’ experiences and perceptions of the MHC model, alongside how mental health services integrate with primary care and the wider community.

4 Implementation

- Quantitative assessment- provider level
  We will evaluate the determinants and consequences of organizational readiness for implementation.
- Qualitative assessment- provider and community levels
  In the absence of a standardized protocol for implementation, fidelity will be explored qualitatively. We will capture operational processes, resource allocation, and center-specific barriers and enablers to implementation. In addition to that, collaboration with the community. We will share the findings with the MH4U team, providers, and community stakeholders to facilitate the translation of evidence into actionable insights.

5 Maintenance

- Quantitative and qualitative assessment- patient, provider, and community levels
  It will be assessed as part of a future follow-up phase ≥ six months after the second phase. We will examine the sustained impact and institutionalization of MHCs, as well as disseminate the findings.

**Table 1:** Reach key indicators, data sources, and target group.

Key indicators	Data source	Target group (level)
% of eligible community members who received at least one MHC service vs. MAP studies	Center records	Patients
Representatives of patients: Distribution of demographic and clinical characteristics vs. MAP studies % of patients referred through each source (self, GP, etc.)	Baseline assessment questionnaire	Patients
Key barriers and facilitators to access the service	Interviews	Patients

**Table 2:** Effectiveness key indicators, data sources, and target group.

Key indicators	Data source	Target group (level)
Mean change in patients outcome over time	Posttraumatic Stress Disorder Checklist (PCL-5), Patient Health Questionnaire (PHQ-9), PHQ-15, Generalized Anxiety Disorder 7 (GAD-7), the Alcohol Use Disorders Identification Test (AUDIT), European Quality of Life 5 Dimensions (EQ-5D-5L) and the visual analogue scale (EQ-VAS), and mental wellbeing by Short Warwick-Edinburgh Mental Wellbeing Scale (SWEMWBS) (22, 35–40).	Patients
Therapeutic alliance	Working Alliance Inventory – Short Revised (WAI-SR) (41).	Patients
Attrition rate: % of patients lost to follow-up Differential retention on demographics and clinical characteristics between completers and dropouts	Center records	Patients
Themes of perceived benefit and challenge	Interviews	Patients

**Table 3:** Adoption key indicators, data sources, and target group.

Key indicators	Data source	Target group (level)
Characteristics of participated MHCs and providers MHC rate of diagnosis and service provision MHC rate of referred patient’s uptake of one or more sessions Center level variability in adoption	Center records	Providers Centers
Team functioning	Team Development Measure (TDM) (42)	Providers
Mental health stigma among providers	Mental Illness Clinicians’ Attitudes Scale (MICA-4) (43)	Providers
Work-related wellbeing and staff health	Copenhagen Psychosocial Questionnaire (COPSOQ-III), Burnout Assessment Tool (BAT), General Health Questionnaire (GHQ-12) and Perceived stress scale (PSS-10) (44–48)	Providers
Themes on organizational barriers and facilitators to adoption	Interviews	Providers
Perceptions and experiences of MHC model in the community	Focus groups	Community stakeholders

**Table 4:** Implementation key indicators, data sources, and target group.

Key indicators	Data source	Target group (level)
% of services delivered as planned Consistence of referral over time per MHC	Center records	Providers Centers
organizational readiness	Organizational Readiness for Implementing Change (ORIC) (49)	Providers
Themes describing core implementation components of fidelity, facilitators, and barriers	Interviews	Directors
Themes capturing real world implementation in practice	Interviews	Providers
Quality and effectiveness of collaboration in implementation	Focus groups	Community stakeholders
Documentation and utilization of feedback and iterative learning from workshops	Workshops	Providers, MH4U team and community stakeholders

**Table 5:** Maintenance key indicators, data sources, and target group.

Key indicators	Data source	Target group (level)
Long-term patient mental health outcomes	PCL-5, PHQ-9, PHQ-15, GAD-7, AUDIT, EQ-5D EQ-VAS and SWEMWBS	Patients
Robustness of long-term effects across patient subgroups Long-term attrition and differential retention by patient characteristics or clinical condition	Center records	Patients
Center-level adaptations post-project Organizational-level sustainability	Interviews	Providers
	Focus groups	Community stakeholders
Engagement in planning and implementing long-term sustainability of MHC practices	Workshops	Providers, MH4U team and community stakeholders

### LHS operationalization

The LHS will be operationalized through staged feedback loops aligned with our study phases to MH4U coordination, MHC leadership and community stakeholders.

1. We will provide direct feedback following the initial and scale up phases and then again after the follow up phase.
2. We will conduct workshops after the scale up phase to share our findings and reflect on implementation challenges and adaptation priorities in real life situation.
3. We will hold a second round of workshops following the follow up phase to present our longitudinal findings, assess sustainability and adaptation after our feedback and support national expansion of the MHC model.

Learning will be operationalized as documented adaptations linked to evaluation findings. Moreover, changes in key RE-AIM indicators between first phases and follow up phase will be examined to assess whether feedback-informed adaptations are associated with improvements in Reach, Effectiveness, Adoption, or Implementation. Workshop documentation will further explore provider and stakeholder perceptions of how feedback influenced decision-making and practice change.

### Data collection methods

All study materials not originally available in Ukrainian underwent forward and backward translation to ensure linguistic and conceptual equivalence. Quantitative validated Ukrainian instruments will be used when available; otherwise, translated tools were pretested, piloted, and adapted as needed and reviewed by the study team at Sumy State University in Ukraine. In addition, we will assess internal consistency reliability and construct validity, as the range of instruments used allows. Then, we will examine indices such as Cronbach’s alpha and compare expected and observed correlations. We will compare these results with published psychometric validations from other countries.

#### Quantitative data collection

##### MHCs

Annual center administrative records, collected routinely at the end of each calendar year, will be obtained retrospectively in January 2026 to capture data from 2025.

##### Patient

Eligible patients for the cohort will receive a flyer during their regular visit at the MHC, containing essential study information with a QR code directing them to a Research Electronic Data Capture (REDCap) registration form (26, 27). We will assess the eligibility based on the submitted registration data. Eligible participants will then be contacted via their preferred way of communication and provided with a personalized link to provide informed consent and complete the self-administered quantitative assessment instruments. Follow-up assessments will be conducted via REDCap at three- and six-month intervals after baseline.

##### Provider

All providers affiliated with the participating MHCs and MHCs that are not supported by MH4U will be invited to participate through a study summary along with a personalized Redcap link, through which they can provide informed consent and complete the quantitative assessment instruments. All providers will be invited again to complete the same set of instruments at the follow-up, which will be approximately one year from the baseline.

#### Qualitative data collection

##### Patient

We developed interview guides for patients structured around the concept of the “patient journey,” and designed to map the individual’s pathway into, through, and beyond the MHCs. We will conduct the interviews by telephone.

##### Provider

We developed interview guides for providers, and they will be interviewed in person during site visits by the Sumy study team.

##### Interview piloting and refinement

Interview guides were piloted in person at Polyclinic No. 1 in Lviv with twelve patients and three providers through in-person interviews to ensure clarity, cultural appropriateness, and alignment with the study objectives. We documented key findings and observational insights. Based on this report, the guides were iteratively refined. The interview lasted approximately 60 min for both patients and providers.

##### Community stakeholders

We developed the focus group guides. One focus group for medical and one for non-medical community stakeholders will be conducted in person per oblast. In Lviv, where the number of MHCs is relatively large, a total of four focus groups will be conducted to ensure representation while maintaining manageable group sizes and discussion dynamics.

##### Non-response and attrition management

We will employ multiple engagement strategies. If a participant does not respond within ten days, we will send a reminder. We will monitor participation patterns and initiate direct follow-up by telephone for participants who remain unresponsive. When available, reasons for non-response or dropout will be recorded.

### Data analysis

#### Quantitative data analysis

We will analyse quantitative data with the statistical computing software R. The analytical approach will be guided by the RE-AIM framework (Tables 1–5).

##### Data preparation and descriptive analyses

Patient, provider, and center level characteristics will be summarized using descriptive statistics (means ± SD or medians [IQR] for continuous variables; counts and percentages for categorical variables) overall, stratified by center, and by center type. All validated instruments will be scored according to their standard scoring protocols.

##### Missing data, attrition, and loss to follow-up

We will quantify attrition rates and assess whether loss to follow up differ by key demographic and clinical characteristics. To reduce selection bias due to differential follow-up, we will apply inverse probability of censoring weighting (IPCW) in longitudinal analyses.

##### Patients

Longitudinal patient outcome data and demographics questionnaires will be analysed and compared to data from the MAP studies. MAP cohort differs at baseline from patients receiving MHCs care. Consequently, we will use inverse probability weighting (IPW) to improve comparability. The IPW model will include key covariates (e.g., age, sex, baseline symptom severity, and prior mental health service use), creating a pseudo-population in which these covariates are balanced between groups. Weighted analyses will be used for all primary MAP vs. MHC outcome comparisons. Additionally, sensitivity analyses will be carried, restricting the sample of MAP subjects to those seeking care and/or with a comparable baseline outcome levels.

We will conduct these analyses on 2 primary cohorts:

1 Retained in care cohort (per protocol style):

This cohort includes patients who remained engaged in MHC care and completed baseline, follow up 1 and 2.

2 Care initiators cohort (intention to treat style):

This cohort will include all enrolled participants regardless of adherence or continued engagement.

For both cohorts, we will estimate intervention effects using DiD approach, comparing pre–post changes in outcomes between MHCs and the MAP cohort, depending on outcome availability and harmonization. Regression models will include center fixed effects and time fixed effects, with the DiD estimand given by the treatment-by-post (or treatment-by-time) interaction. Where multiple follow-ups are available, we will implement an event-study specification (baseline, follow-up 1, follow-up 2). We will obtain 95% confidence intervals, and *p*-values via center-level wild cluster bootstrap (1,000 draws). As a robustness check for small-sample inference, we will also report results using a Kenward–Roger/Satterthwaite-style degrees-of-freedom adjustment. For retained in care cohort, we will adjust the analyses using inverse probability of censoring weighting (IPCW) to address differential loss to follow up.

##### Providers

Provider outcomes will first be summarized descriptively at baseline from participating MHCs and compared with outcomes from MHCs that are not supported by MH4U. For prospectively collected survey outcomes, we will evaluate changes from baseline to follow-up using a DiD framework (pre–post change in MH4U supported vs. non-supported centers), with center-clustered inference.

Integration with the LHS approach, all analytic results will be shared at each phase with the MH4U team, providers, and community stakeholders via collaborative workshops and direct feedback (21).

#### Qualitative data analysis

We will conduct all qualitative assessments in Ukrainian with recorded audio. Verbatim transcripts will be analysed in Ukrainian to preserve the linguistic and cultural nuances of participants’ responses. All analyses will be conducted in MAXQDA (2024). Coding will combine deductive and inductive strategies. Deductive coding according to the interview and focus group guides, inductive codes will emerge during the content analyses. We aim to enhance analytic trustworthiness, reduce bias, and improve code clarity. Accordingly, two researchers at the University of Zurich and two at Sumy State University will independently analyse a subset of three transcripts. Both teams will iteratively refine the codebook, reconcile differences, and document decisions transparently. We will employ a structured content analysis approach, based on the study’s research questions. Two experienced qualitative researchers from Sumy State University will lead the main analyses. We will conduct mid-term analyses after every 4th patient interview within each center to determine content saturation. Data collection will continue until content saturation is reached and no new information emerges.

#### Mixed methods integration

First, we will analyse quantitative and qualitative data separately. Then, we will integrate findings from both data using a convergent mixed methods design, enabling triangulation across sources. Integration will involve comparing results of both analyses to identify convergencies (agreement), complementarities (different but non-contradictory findings), divergencies (conflicting findings) and expansions (findings that overlap but still provide additional interpretation) (28, 29). This approach will deepen understanding into the MHCs model by providing validation and a holistic view of whether numerical trends match with participants lived experiences.

#### Data management

We will collect informed consent and quantitative data from patients and providers via the data management system redcaps (26, 27). REDCap is provided by Epidemiology, Biostatistics, and Prevention Institute (EBPI), University of Zurich (UZH), and hosted at ETH Zurich on Leonhard Med (Lamed). REDCap accounts will be created for authorized research group members.

Regarding qualitative data, we will record provider and patient interviews as well as focus groups, and the recordings will include verbal consent at the beginning of each session. We will ask participants not to share personal information (e.g., name, address, birth date). Recording will be transcribed verbatim by an external transcription provider (Transcript or application) and imported into the software MAXQDA for qualitative data analysis (30).

#### Monitoring and quality assurance

We will implement and maintain quality assurance and quality control with written SOPs to ensure data is generated, documented, and reported in compliance with the study protocol.

#### Confidentiality and coding

All project data will be accessible only to authorized personnel with a defined role in the study.

All quantitative and qualitative data will be stored on UZH servers separately in two different files: one containing identifiable information only (participant identification list) and another with pseudonymized (coded) data for analyses. Participants will be assigned a unique study identifier (ID). Data analyses will only be conducted using pseudonymized datasets.

We will create a participants’ identification list, containing the personal data together with participants’ IDs. This list will be saved password-protected on a secure server at the UZH for 10 years. No identifying information will appear on any of the study’s written (scientific publications, conference posters) or verbal (presentations) outputs.

### Ethics and dissemination

The study involving humans was approved by the Commission on Bioethics of Sumy State University, Ukraine (60–236 from 06/11/2024). The study is conducted in accordance with the local legislation and institutional requirements. Participants received modest remuneration in recognition of the time spent for completing study questionnaires.

Study findings will be disseminated through peer-reviewed publications and conference presentations. In line with the LHS approach that underpins this study, findings will also be shared with MH4U and policymakers throughout the project. Pseudonymised data will be made available on reasonable request after the main results have been published.

## Discussion

Evaluation of MHCs in Ukraine will address the evidence gap on transitioning from institutionalization toward a community care model. In a war affected, post-totalitarian context, community mental health care extends beyond service delivery. Ukraine’s modern mental health system remains dominated by biomedical approaches delivered primarily through inpatient psychiatric institutions and care homes (psycho-neurological internats) (18). Furthermore, despite recent efforts to raise awareness about mental health and to reduce stigma, mental health stigma remains a significant and complex challenge (31). Even before the escalation of the war, mental health resources were insufficient to meet adequate population needs (7). Together these factors have reinforced high level of institutionalization, segregation, and stigma, alongside limited availability of psychological, rehabilitative, and community-based services (18). MHCs will lower practical and symbolic barriers to care, by integrating services within communities and emphasizing psychosocial support alongside clinical care (14, 32). Consequently, MHCs have the potential to reduce stigma, strengthen trust in public health institutions, and improve continuity of care for individuals with mental health conditions (14). These dimensions are critical in Ukraine, where repeated exposure to displacement, loss, and insecurity on addition to their early life hardships (33).

Evaluating MHCs in conflict settings can generate transferable evidence for strengthening mental health systems well beyond the direct war exposure within Ukraine. Evidence from the region suggests that the psychological consequences of the conflict extend across borders. A cross-sectional study in Bosnia and Herzegovina demonstrated that the emergence of the Ukraine war is significantly associated with the reactivation of PTSD symptoms among individuals who had lived through the Bosnian war (34). Consistent with this, a multi-country study across 11 European countries reported elevated symptoms of depression, anxiety, and PTSD in populations indirectly exposed to the war, likely due to media exposure, refugee movements, and socioeconomic uncertainty (34). MHCs are likely to operate not only as a response to recent war-related exposure, but also as a model for delivering accessible, sustainable, and community-trusted mental health care in the long run. Findings from this evaluation will provide timely evidence on scalable model capable of addressing both acute and long-term psychological needs under conditions of high demand and limited resources. However, application of this evidence in other countries will require contextual and local adaptation.

Conducting this evaluation in an active conflict setting introduces unique operational and ethical challenges. First, certain mental health topics remain highly sensitive in this context. Careful wording of instruments and flexible consent procedures are adapted to avoid causing psychological distress or re-triggering trauma. Second, ongoing conflict also affects the planned scale-up of the MHC model, introducing uncertainties that cannot be fully controlled. Third, the mixed-methods design adds further complexity. Quantitative and qualitative data must be integrated under tight timelines while providing iterative feedback. This requires sustained communication between MH4U and the research team at Sumy and Zurich universities, as well as standardized data management to ensure timely and reliable feedback.

### Strengths and limitations

#### Strengths

This multidimensional approach, linking patient outcomes with provider and community-level factors, enables the understanding of contextual, organizational, and system-level needs. It fills a critical gap in the existing literature, which has largely focused on clinical outcomes rather than the mechanisms and conditions required for effective transition from institutionalized psychiatric services to the community-based care model. The longitudinal study design using a mixed-methods design, guided by the RE-AIM framework will generate actionable evidence to inform practice and scale up. This study also uses LHS approach with iterative feedback loops and stakeholder engagement are designed to ensure that emerging evidence informs practice and decision-making in real time.

#### Limitations

The variability in implementation across centers. As a result, fidelity cannot be directly measured due to the absence of standardized fidelity indicators tailored to the nature of implementation across every center. In addition, the absence of a formal economic evaluation limits conclusions regarding the cost-effectiveness and financial sustainability of the MHC model at scale.

## Conclusion

The evaluation will generate evidence for MH4U and Ukrainian policymakers for quality improvement and to guide the scale-up of MHCs during ongoing war and recovery. The findings will also provide a transferable lessons for other health care systems seeking to transition from institutionalized to community-based mental health care.
